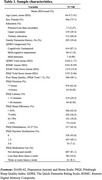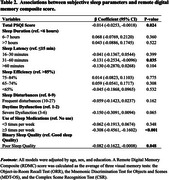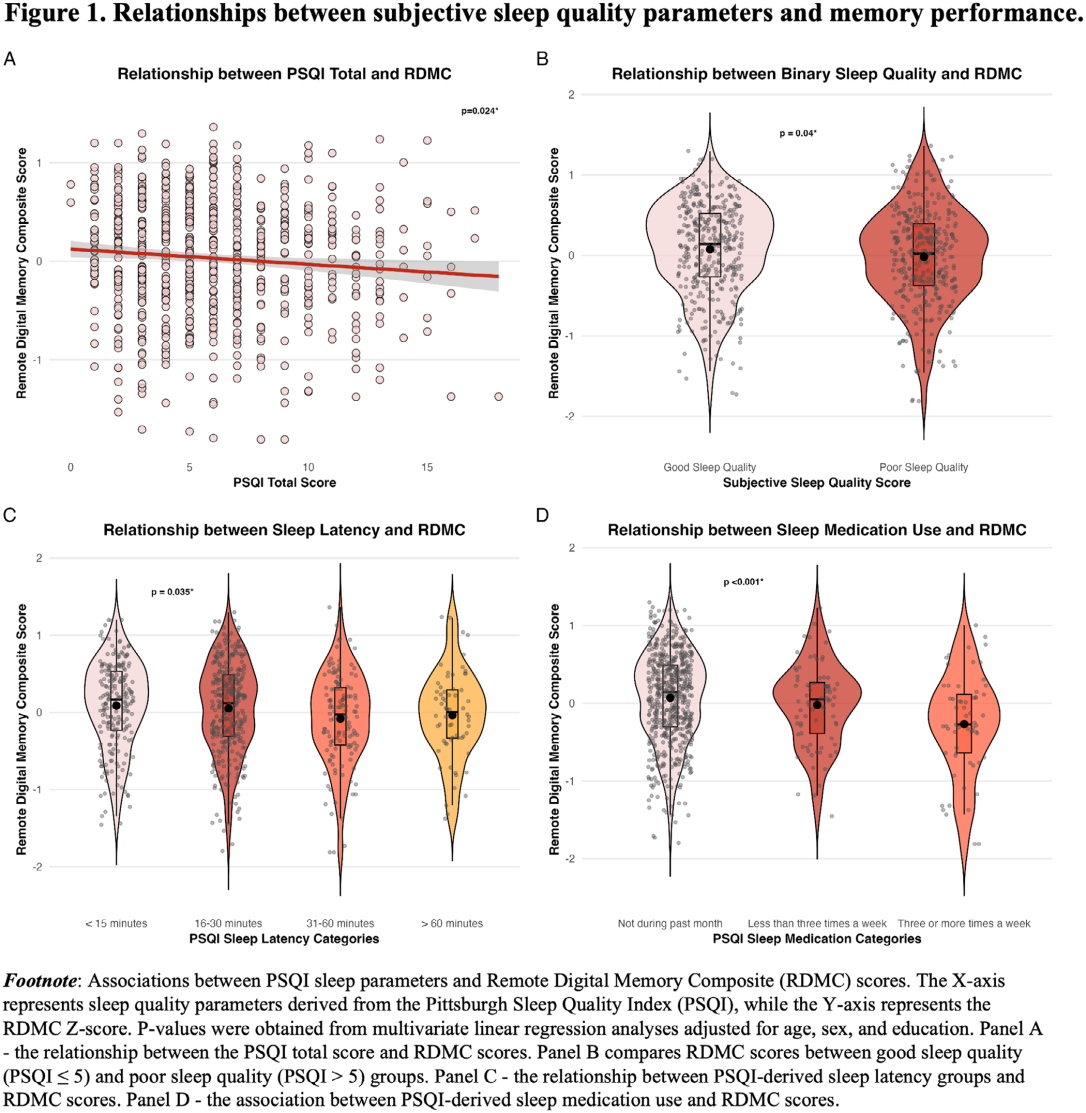# Poor sleep quality is associated with worse memory performance on digital remote cognitive assessment in the population‐based screening study REAL AD

**DOI:** 10.1002/alz70857_103043

**Published:** 2025-12-25

**Authors:** Laura Stankeviciute, Iris Bosch, Fredrik Öhman, Ellen Hanna Singleton, Frida Lenér, Maria Dottori, Kajsa Quitz, Nicolai Franzmeier, Silke Kern, Henrik Zetterberg, Kaj Blennow, Michael Schöll

**Affiliations:** ^1^ Department of Psychiatry and Neurochemistry, Institute of Neuroscience and Physiology, Sahlgrenska Academy, University of Gothenburg, Gothenburg, Västra Götalands, Sweden; ^2^ Department of Public Health and Community Medicine, Sahlgrenska Academy, University of Gothenburg, Gothenburg, Sweden; ^3^ Department of Psychiatry and Neurochemistry, Institute of Neuroscience and Physiology, The Sahlgrenska Academy at the University of Gothenburg, Mölndal, Västra Götalands län, Sweden; ^4^ Department of Psychiatry and Neurochemistry, University of Gothenburg, Mölndal, Västra Götalands län, Sweden; ^5^ Wallenberg Centre for Molecular and Translational Medicine, University of Gothenburg, Gothenburg, Sweden; ^6^ Region Västra Götaland, Sahlgrenska University Hospital, Department of Neuropsychiatry, Gothenburg, Västra Götalands län, Sweden; ^7^ Region Västra Götaland, Sahlgrenska University Hospital, Department of Neuropsychiatry, Gothenburg, Västragötalandsregionen, Sweden; ^8^ Region Västra Götaland, Research, Education, Development & Innovation (REDI), Primary Health Care, Gothenburg, Sweden; ^9^ Region Västra Götaland, Research, Education, Development & Innovation (REDI), Primary Health Care, Gothenburg, Västra Götalands län, Sweden; ^10^ Närhälsan Primary Health Care, Region Västra Götaland, Gothenburg, Sweden; ^11^ Munich Cluster for Systems Neurology (SyNergy), Munich, Bavaria, Germany; ^12^ Institute for Stroke and Dementia Research (ISD), LMU University Hospital, LMU, Munich, Bavaria, Germany; ^13^ Region Västra Götaland, Sahlgrenska University Hospital, Department of Neuropsychiatry, Gothenburg, Sweden; ^14^ Department of Psychiatry and Neurochemistry, Institute of Neuroscience and Physiology, the Sahlgrenska Academy, University of Gothenburg, Molndal, Sweden; ^15^ Department of Psychiatry and Neurochemistry, University of Gothenburg, Mölndal, Sweden; ^16^ Dementia Research Centre, Queen Square Institute of Neurology, University College London, London, United Kingdom

## Abstract

**Background:**

Sleep disturbances are prevalent in Alzheimer's disease (AD), often emerge in preclinical stages, and may act as risk factors for exacerbating AD pathology and accelerating disease progression. Poor subjective sleep quality has been associated with more abnormal AD biomarkers and poorer cognitive performance in cognitively unimpaired (CU) older adults. However, evidence from large‐scale population‐based studies integrating novel remote digital cognitive assessments (DCAs), blood‐based biomarkers (BBBs) of AD and sleep measures remains scarce. Therefore, we explored whether worse subjective sleep quality is associated with worse DCA scores in older adults.

**Method:**

The REAL AD study is a population‐based screening cohort aimed at validating the combined use of BBB and DCA for early AD detection. Of the *N* = 1634 participants who completed the Pittsburgh Sleep Quality Index (PSQI) and the *N* = 1345 who performed remote digital memory assessments via the neotivTrials app, the final analysis included 748 (mean age=64, 76% female, 87% CU, Table 1) participants, following the quality control procedures. A Remote Digital Memory Composite (RDMC) score was calculated as the average of three visual memory tests: the Object‐in‐Room Recall Test, Mnemonic Discrimination Test for Objects and Scenes, and Complex Scene Recognition Test. Associations between PSQI components (e.g., total score, duration and sleep latency) and RDMC were examined using linear regression models, adjusted for age, sex, and education. Sensitivity analyses included additionally correcting for depression and anxiety symptoms (DASS‐42).

**Result:**

Poor subjective sleep quality (PSQI Total Score) was associated with lower RDMC performance (*p* = 0.024) (Table 2. Figure 1). Prolonged sleep latency (31–60 minutes) and frequent sleep medication use (≥3 times per week) were significantly associated with lower RDMC scores (*p* = 0.04, *p* < 0.001, respectively). These associations were attenuated after adjustment for DASS‐42.

**Conclusion:**

In this population‐based study, subjective sleep quality parameters were associated with worse memory performance on digital cognitive tests. These results highlight the interplay between cognition and sleep in preclinical AD. Ongoing analyses leveraging relevant BBB measures and objective sleep assessment aim to expand these preliminary findings, providing insights into the biological mechanisms and the value of integrating digital health technologies with biomarker‐based approaches for early AD detection.